# Synthesis and evaluation of amino acid ionic liquid for enhanced oil recovery: experimental and modeling simulation studies

**DOI:** 10.1038/s41598-025-85560-8

**Published:** 2025-01-16

**Authors:** E. M. Mansour, R. Hosny, Ammona S. Mohamed, Fatma M. Abdelhafiz

**Affiliations:** 1 PVT Lab, Production Department, Egyptian Petroleum Research Institute, 1 Ahmed El Zomor St., Nasr City, Cairo 11727 Egypt; 2 PVT Services Center, Egyptian Petroleum Research Institute, 1 Ahmed El Zomor St., Nasr City, Cairo 11727 Egypt; 3 EOR Lab., Production Department, Egyptian Petroleum Research Institute, 1 Ahmed El Zomor St., Nasr City, Cairo 11727 Egypt; 4 Core Lab Center, Egyptian Petroleum Research Institute (EPRI), 1 Ahmed El Zomor St., Nasr City, Cairo 11727 Egypt; 5 Petrochemicals Department, Egyptian Petroleum Research Institute, 1 Ahmed El Zomor St., Nasr City, Cairo 11727 Egypt

**Keywords:** Chemical enhanced oil recovery, Ionic liquid, Surface activity, Critical micelle concentration, Interfacial tension, Wettability alteration, Reservoir simulations, Chemical engineering, Energy

## Abstract

Recovering the remaining oil after primary and secondary extraction methods poses a significant challenge. Enhanced oil recovery (EOR) techniques, which involve injecting fluids into reservoirs, aim to increase recovery rates. Ionic liquids, known for their adaptability, are emerging as promising agents in EOR, improving oil displacement by reshaping fluid properties and interacting with reservoir rocks. This study investigates the eco-friendly amino acid ionic liquid, AAIL [G0.5 C12][Pro], for EOR applications, focusing on its characterization and performance. Using pre-prepared quaternary ammonium salt PAMAM G0.5 C12 and proline, AAIL [G0.5 C12][Pro] was synthesized and confirmed via FTIR and 1H-NMR analyses. Rheological analysis identified 7 g of AAIL [G0.5 C12][Pro] as the optimal concentration for peak performance. Laboratory sand-pack displacement experiments demonstrated an 11% increase in oil recovery at this concentration. Further, a 3D reservoir model simulation validated the enhanced oil recovery potential of AAIL [G0.5 C12][Pro]. The study introduces the novel amino acid ionic liquid [G0.5 C12][Pro], which demonstrates superior effectiveness in enhancing oil recovery through significant wettability modification and interfacial tension reduction, underscoring its potential as an effective and environmentally friendly EOR agent compared to other ionic liquids and conventional methods.

**Supplementary Information:**

The online version contains supplementary material available at 10.1038/s41598-025-85560-8.

## Introduction

Oil flooding is a common technique in the petroleum industry, especially in enhanced oil recovery (EOR), and aims to maximize the extraction of crude oil from reservoirs^1,2^. After primary recovery, driven by natural pressure or pump-assisted methods, and secondary recovery, typically using water flooding, a significant amount of oil remains trapped in the reservoir due to factors like capillary forces, poor permeability, or unfavorable fluid-rock interactions^3,4^. To overcome these challenges, tertiary recovery methods, known as chemical EOR, are employed to modify reservoir conditions and improve oil mobilization^5^. Oil flooding through water, chemical, and gas injection plays a vital role in these methods^6,7^.

Chemical flooding builds on water flooding by introducing chemical agents such as surfactants, polymers, or alkaline solutions to alter the interactions between oil, water, and reservoir rocks^8^. Surfactants reduce the interfacial tension (IFT) between oil and water, allowing oil to be more easily displaced, while polymers enhance water viscosity, improving the efficiency of the flood^9^. Alkaline flooding involves injecting chemicals that react with acidic components in the crude oil, creating surfactants in situ, further reducing IFT and aiding in oil recovery^10^.

Among the chemical agents used in oil flooding, ionic liquids (ILs) have emerged as a versatile and promising class of chemicals for EOR^11,12^. ILs are salts that remain liquid at relatively low temperatures, often below 100 °C. They consist of organic cations and inorganic or organic anions, and their properties can be tailored for specific applications^13,14^. ILs are gaining attention in oil recovery due to several advantages^15–18^. They are effective in reducing IFT between oil and water, which facilitates the mobilization of trapped oil in the reservoir pores, reducing residual oil saturation^19,20^. ILs also alter the wettability of reservoir rocks, shifting conditions from oil-wet to water-wet, which enhances the displacement of oil by injected fluids^21–23^. Their thermal stability makes them suitable for use in both conventional and high-temperature reservoirs, and their ability to dissolve a variety of compounds aids in oil solubilization and mobility^23–27^.

Research has shown that ILs can significantly improve oil recovery through various mechanisms^28–30^. For instance, they can reduce IFT, allowing oil droplets to detach from rock surfaces and flow through the porous medium^31^. Additionally, certain ILs modify the wettability of reservoir rocks, enabling more water to push oil toward production wells^32^. At critical micelle concentrations (CMC), ILs can form micelles that encapsulate oil droplets, preventing re-adhesion to rock surfaces and facilitating their transport^33^. Some IL formulations also increase the viscosity of the flooding fluid, improving sweep efficiency and preventing the bypassing of oil-rich zones within the reservoir^34^.

The emergence of eco-friendly and sustainable ionic liquids (ILs), particularly amino acid ionic liquids (AAILs) derived from naturally occurring amino acids, is increasingly prominent in enhanced oil recovery (EOR)^35^. AAILs are advantageous due to their environmentally benign nature while still possessing essential properties for EOR, such as thermal stability and surface activity^36^. These ionic liquids can be categorized by their functional groups, significantly affecting their characteristics and applications in EOR^37^. For example, lysine-based ionic liquids, derived from amino acids with amine functional groups, exhibit strong surface activity and improve wettability alteration^38^. Similarly, glutamate-based ionic liquids, which incorporate the carboxylic acid group from glutamic or aspartic acid, show excellent solubility and effective reduction of interfacial tension^39^. Serine-based ionic liquids featuring hydroxyl groups enhance interactions with water and oil, boosting oil recovery efficiency^40^. Additionally, cysteine-based ionic liquids, utilizing the sulfhydryl functional group, facilitate oil solubilization and the extraction of heavy crude oils^41^. Tyrosine-based ionic liquids, characterized by their phenolic structures, are known for their strong surface activity and capability to stabilize emulsions, further aiding oil recovery processes^39^. Quaternized amino acid ionic liquids, synthesized from amino acids combined with quaternary ammonium salts, enhance surfactant properties and improve oil displacement in EOR applications^42^.

This study aims to synthesize and evaluate a novel amino acid ionic liquid, AAIL [G0.5 C12][Pro], for its potential to enhance oil recovery. By conducting laboratory experiments and simulation modeling, this research endeavors to advance the application of ionic liquids by developing an environmentally sustainable, effective solution under real-world reservoir conditions. It builds on previous advancements while addressing the limitations of existing ionic liquids, such as their reduced efficacy in extreme environments and the necessity for greener alternatives. Ultimately, this work positions AAIL [G0.5 C12][Pro] as a promising candidate for significantly improving oil recovery while minimizing environmental impact. This study focuses on the dual mechanisms of AAIL [G0.5 C12][Pro] in EOR applications, namely moderate interfacial tension (IFT) reduction and significant wettability alteration.

## Materials and experimental

### Materials

Polyamidoamine (PAMAM, G_0.5_ C_12_) was prepared in previous work^43^, Amino acid (Proline 99%) was purchased from Aldrich (Sigma-Aldrich), and Ethyl alcohol (EtOH 99%) was purchased from ADWIC (Qaliubiya, Egypt).

### Methodology for synthesis

#### Synthesis of amino acid ionic liquid AAIL [G0.5 C12][Pro]

**Scheme 1:**
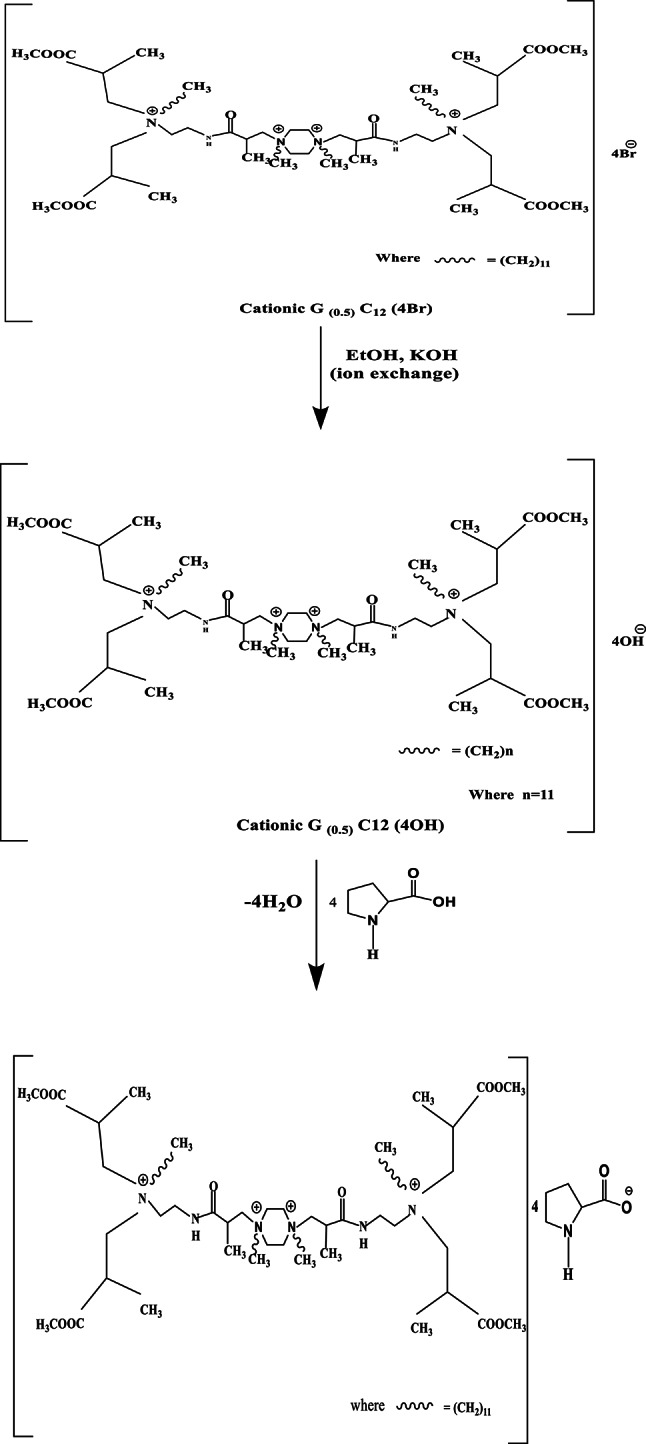
Synthesis of the amino acid ionic liquid (AAIL [G0.5 C12][Pro]).

The amino acid ionic liquid was synthesized via a two-step process: (1) ion exchange and (2) neutralization, as illustrated in Scheme 1. The reaction occurred in a flask fitted with a magnetic stirrer at room temperature for three hours. The mixture was cationic polyamidoamine (PAMAM) (G0.5 C12, 0.1 mol) and potassium hydroxide (KOH, 0.4 mol) in pure ethyl alcohol (EtOH). Upon removing KBr by filtering, the compound ([G_0.5_ C_12_][OH])-Ethanol solution was concentrated through rotation evaporation at 35 °C. By adding a small amount of amino acid (proline) to the concentrated [G0.5 C12][OH] solution and stirring it for 24 h at 40 °C, AAIL [G0.5 C12][Pro] was produced. After rotation evaporation at 50 °C, the residual solution containing the ionic liquid was dried in a vacuum at 60 °C, and the product was obtained. To obtain the pure amino acid ionic liquid at the final stage, ethanol was added to the mixture to precipitate the extra amino acid. To remove ethanol, the solution was rotary evaporated after filtration. Finally, the product AAIL [G0.5 C12][Pro] was obtained by drying at 60 °C with 81% yield^44^, as shown in Scheme 1.

### Structural confirmation of AAIL [G0.5 C12][Pro]

The chemical structure of synthesized AAIL [G0.5 C12][Pro] was verified by The Fourier Transform Infrared (FTIR) and Proton Nuclear Magnetic Resonance (^1^HNMR) spectra.

The Fourier Transform Infrared analysis was used using the ATI Mattson Genesis and FTIR spectrophotometer. The spectra were obtained with a resolution of 2 cm^−1^, employing an angle of incidence of 80°. For the FT-IR measurement of AAIL [G_0.5_ C_12_][Pro], an ethanol solution and a disk made of KBr were used. 0.5 g KBr and 5 mg dry samples were mixed in a mortar, and then, the whole ground pressure was applied at 1.5 bars for 1 min. The test was conducted at room temperature with a 400 ~ 4000 cm^−1^. The measurement was carried out at the Egyptian Petroleum Research Institute.

The Proton Nuclear Magnetic Resonance (^1^HNMR) spectra for the synthesized AAIL were determined in DMSO at room temperature using the NMR spectrometer, Jeol ECA, Japan, at the National Research Center.

### Critical micelle concentration and surface surface tension at critical micelle concentration (γ_cmc_)

The CMC value was calculated using surface tension measurements. Solutions of AAIL [G0.5 C12][Pro] were prepared at different concentrations and measured using the Attention Theta Optical Tensiometer. The surface tension was plotted against the logarithm of the concentration, and the CMC was determined at the curve’s inflection point. The cmc value was calculated using AAIL [G0.5 C12][Pro] solutions via surface tension technique using Attention Theta Optical Tensiometer (Biolin Scientific Company, Finland). Different surface parameters of the synthesized AAIL were detected, including effectiveness, maximum surface excess, minimum surface area, and standard micellization and adsorption-free energies**.**

Ionic liquids with long aliphatic chains that also work as surfactants are an exciting type of surface-active substances for use in catalysis and synthesis^45^. At specific concentrations, amphiphile molecules tend to aggregate into micelles, vesicles, bilayers, and a variety of various nanostructures in a variety of media^46^. Critical micelle concentration (*cmc*), maximum surface excess (*Γ*_*ma*x_), minimum surface area (A_min_), thermodynamic values, and other micellization parameters are essential in various applications.

In the petroleum industry, cmc is considered before injecting ionic liquid into a reservoir for enhanced oil recovery (EOR) application. Below the *cmc* point, the interfacial tension between the oil and water phase is no longer effectively reduced^47^. If the concentration of the ionic liquid is kept a little above the *cmc*, the additional amount covers the dissolution with existing brine in the reservoir. It is desired that the amino acid ionic liquid will work at the lowest interfacial tension (IFT).

#### Effectiveness (***π***_***cmc***_)

The efficacy of a compound in reducing surface tension is quantified by the maximum surface pressure (*π*_*cmc*_), as determined by the following equation (Eq. 1):

1 $$\pi_{{{\text{cmc}}}} = \gamma_{{\text{o}}} - \gamma_{{{\text{cmc}}}}$$

where γ_o_ and γ_cmc_ are the surface tension of pure water and surface tension at cmc, respectively^48^.

The most efficient surfactant results in a more significant lowering of surface tension at *cmc.* Compound AAIL [G0.5 C12][Pro] had the most significant reduction in surface tension at its *cmc* value, according to the values of *πcmc* shown in Table 1.

**Table 1:** cmc, γcmc, πcmc, Γmax, Amin and ΔG_mic_, ΔG_ads_ of the synthesized.

Compounds	cmc × 10^–2^ (mol/l)	γ_cmc_ (mN/m)	Π_cmc_ (mN/m)	Γ_max_ × 10^–11^ (mol/cm^2^)	A_min_ (nm^2^)	ΔG_mic_ kj/mol	ΔG_ads_ kj/mol
[G0.5 C12]	0.020	28.69	43.30	2.83	5.86	− 21.10	− 21.10
AAIL [G0.5 C12][Pro]	0.0128	26.52	45.48	5.07	2.89	− 22.21	− 22.3

[G0.5 C12] and AAIL [G0.5 C12][Pro] at 25 °C.

#### Maximum surface excess (Γ _max_)

The evaluation of Γ max for ions was conducted using the Gibbs adsorption equation^49^, derived from the slope of the surface tension plot (δγ/δ log c) below the critical micelle concentration (*cmc*) according to Eq. 2:

2 $$\Gamma_{\max } = - \frac{{\delta_{\gamma } }}{\delta \log c} \frac{T}{2.303nRT}$$

Here, Γmax represents the maximum surface excess of ion concentration, R is the gas constant, T is the absolute temperature, $\frac{{\delta_{\gamma } }}{\delta \log c}$ is the slope of the γ vs. -log C^50^.

The utility of ionic liquids at various interfaces relies on their adsorption capacity. The disparity in packing density among differenionic liquids elucidates the alteration in surface activity at the air/water interface, expressed through (*Γ *_*max*_) and (*A*_*min*_). The surface excess concentration value increases as the surface area decreases^51^, as illustrated in Table 1. The values of *Γ *_*max*_ highlight the impact of the surface tension plot slope (δγ/δ log c) on the maximum surface excess.

#### Minimum surface area (A_min_)

The degree of packing and orientation of the adsorbed molecule can be determined from the area per molecule at the interface.

Each adsorbed molecule at the interface occupies the average minimum surface area (*A*_*min*_, measured in square angstroms)^52^. It is determined by the following equation (Eq. 3):

3 $$A_{\min } = \, 10^{16} /N_{A} \cdot \Gamma_{\max }$$

N_A_ is Avogadro’s number and Γ_*max*_ (mol m^−2^) is the maximum surface excess of adsorbed molecules at the interface.

Decreasing Γ_*max*_ indicates fewer adsorbed molecules at the air/water interface. Hence, the area available for each molecule at the interface will increase as shown from Amin’s data in Table 1.

The results indicated that the molecular structure of the AAIL facilitated its swift adsorption at the air–water interface, enabling efficient self-orientation and decreasing surface tension.

##### IFT measurements

In the interfacial tension (IFT) measurements, the pendant drop method was employed to determine the IFT between the synthesized amino acid ionic liquid (AAIL [G0.5 C12][Pro]) and crude oil. Various concentrations of the AAIL solution in brine (ranging from 1 g/L to 11 g/L) were prepared, and measurements were conducted at room temperature. A drop of crude oil was injected into the brine solution containing the AAIL, and its deformation was monitored over time. The IFT was recorded as the oil drop adjusted in the ionic liquid solution. This method was essential for optimizing conditions in enhanced oil recovery by minimizing the IFT between the oil and water phases.

#### Contact angle measurement

Wettability is assessed by measuring the contact angle between the fluid and the rock surface, directly influencing oil recovery efficiency. Contact angle measurements were conducted using varying concentrations of the amino acid ionic liquid [G0.5 C12][Pro] in the presence of crude oil. Rock samples were cleaned and conditioned before being treated with different concentrations of [G0.5 C12][Pro]. After treatment, crude oil droplets were placed on the rock surfaces, and the contact angle was measured using the static sessile drop method with a goniometer. Measurements were performed at ambient temperature, with multiple readings for each concentration to ensure accuracy and reproducibility.

#### Thermodynamic properties of the prepared AAIL

The micellization and adsorption free energy at the interfaces have been calculated using surface tension measurements. The adsorption of AAIL molecules at the interface under equilibrium conditions reduces surface tension. The Gibbs adsorption equation provided the number of molecules adsorbed at the interface per unit area. The *cmc* values play a vital role in calculating *ΔG*_*mic*_*.* Standard free energies of micellization and adsorption (*Δ*G^o^_*mic*_, *Δ*G^o^_*ads*_) were computed using Gibbs adsorption rules^53^ As follows (Eqs. 4 & 5):

4 $$\Delta {\text{G}}^{{\text{o}}}_{{{\text{mic}}}} = {\text{n RT ln}}cmc$$

5 $$\Delta {\text{G}}^{{\text{o}}}_{{{\text{ads}}}} = \Delta {\text{G}}^{{\text{o}}}_{{{\text{mic}}}} - { 6}.0{23} \times { 1}0^{{ - {1}}} \times \, \pi_{{{\text{cmc}}}} \times {\text{ A}}_{{{\text{min}}}}$$

where R is the gas constant, and T is the absolute temperature.

A negative *ΔG*_*mic*_ value signified the generation of micelles within the solution’s bulk phase. A negative *ΔG*_*mic*_ value suggests that micellization, a spontaneous process of association and dissociation, enables AAIL molecules to be adsorbed at the interface. Concurrently, the negative value of ΔGmic increases the solvent’s free energy, compensating for the tendency of AAIL molecules to adsorb on surfaces and interfaces before and during micelle formation. The negative value of ΔGads indicates the spontaneous behavior of the molecule adsorption at the air–liquid interface*.* Based on the micelle aggregation and adsorption capabilities of AAIL, it is expected that they it contribute to use in oil solubilization and displacement processes in the enhanced oil recovery field.

### Rheological measurements

Rheometer (Modular Compact Rheometer: MCR 102e) is used for different types of fluids (crude oils, polymer solutions, surfactants,…etc.) at pressure till 150 bar and temperature till 250 °F to determine rheological properties of these samples at different conditions. The representative samples were measured according to ASTM D-2196^54^.

### Sand-packed model—flooding test

**Fig. 1:**
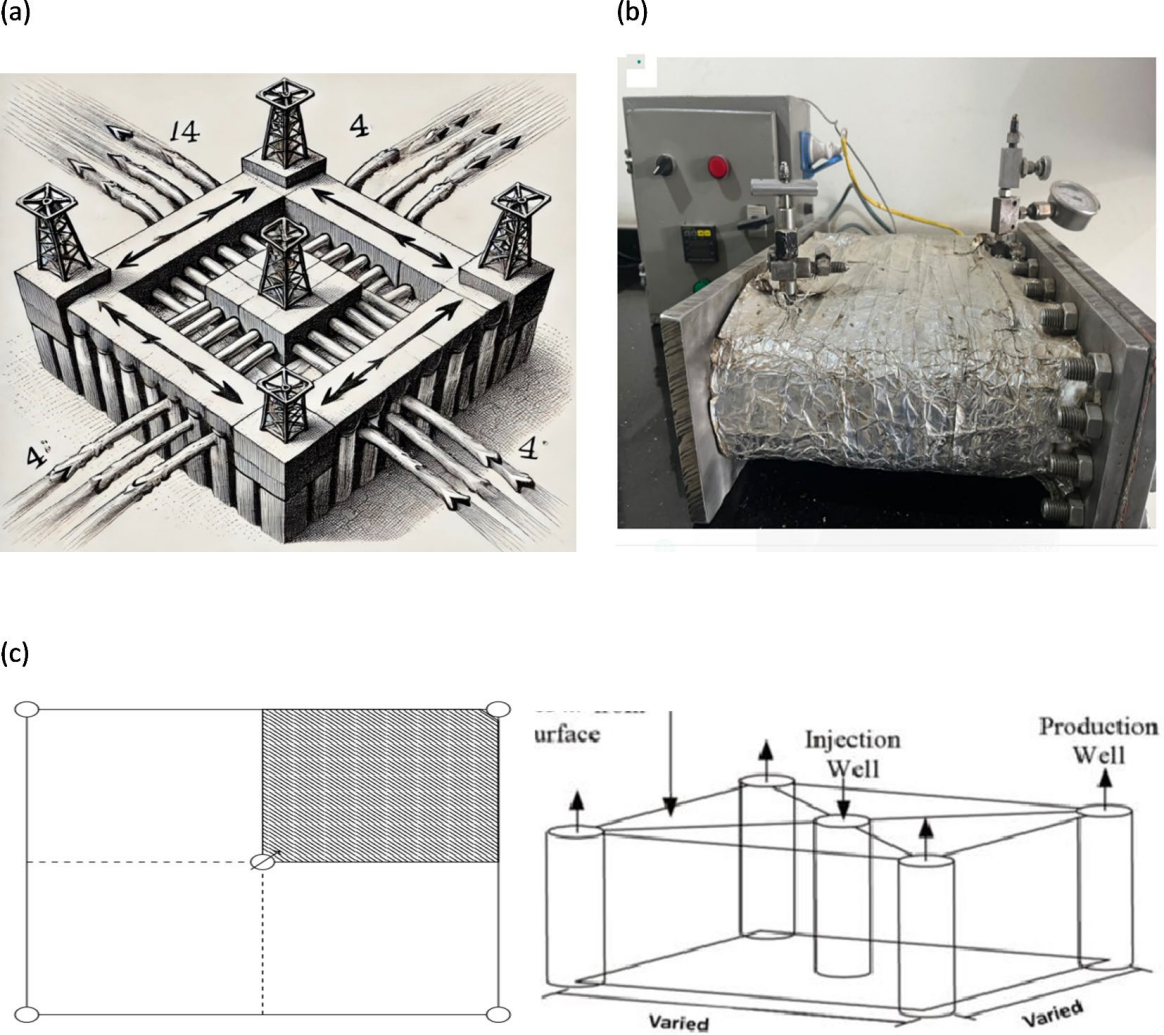
Images of the 1/4 Five-spot model.

The experimental procedure for the sand-packed 2-D model (1/4 Five-spot) flooding test involves preparing a column filled with tightly packed sand to simulate the porous structure of an oil reservoir. The 1/4 Five-spot setup used in oil recovery involves four injection wells at the corners of a square, with a production well located at the center, as shown in Fig. 1a, where the model’s core is filled with sand to simulate a porous medium, similar to reservoir rock. The sand must be packed uniformly to simulate flooding scenarios during the reservoir production cycle, following established procedures outlined in previous literature^2^. This includes washing and evacuating the sand, saturating it with brine, soaking it with oil, and then flooding it with brine until the oil cut ceases. In the 1/4 setup, only one quadrant is typically modeled, featuring one injection point and one production point on opposite sides, as illustrated in the laboratory work diagram in Fig. 1b and the schematic diagram in Fig. 1c. Arrows indicate the movement of injected water from the outer wells toward the central production well, driving the oil toward the middle for efficient extraction as secondary recovery. This method also helps to enhance oil recovery by maintaining pressure and directing oil flow toward the production point by injecting various AAIL [G0.5 C12][Pro] solutions at specified reservoir conditions with a temperature of 167°F and a pressure of 1500 psi. Throughout the test, measurements are taken to monitor oil production rates and fluid properties. After completion, the results are analyzed to assess the effectiveness of the AAIL [G0.5 C12][Pro] solution in enhancing oil recovery.

## Results and Discussion

### Characterization of synthesis of AAIL [G0.5 C12][Pro]

The synthesized amino acid ionic liquid (AAIL [G0.5 C12][Pro]) was characterized using FTIR and ^1^H NMR Spectrum.

#### FTIR spectra

**Fig. 2:**
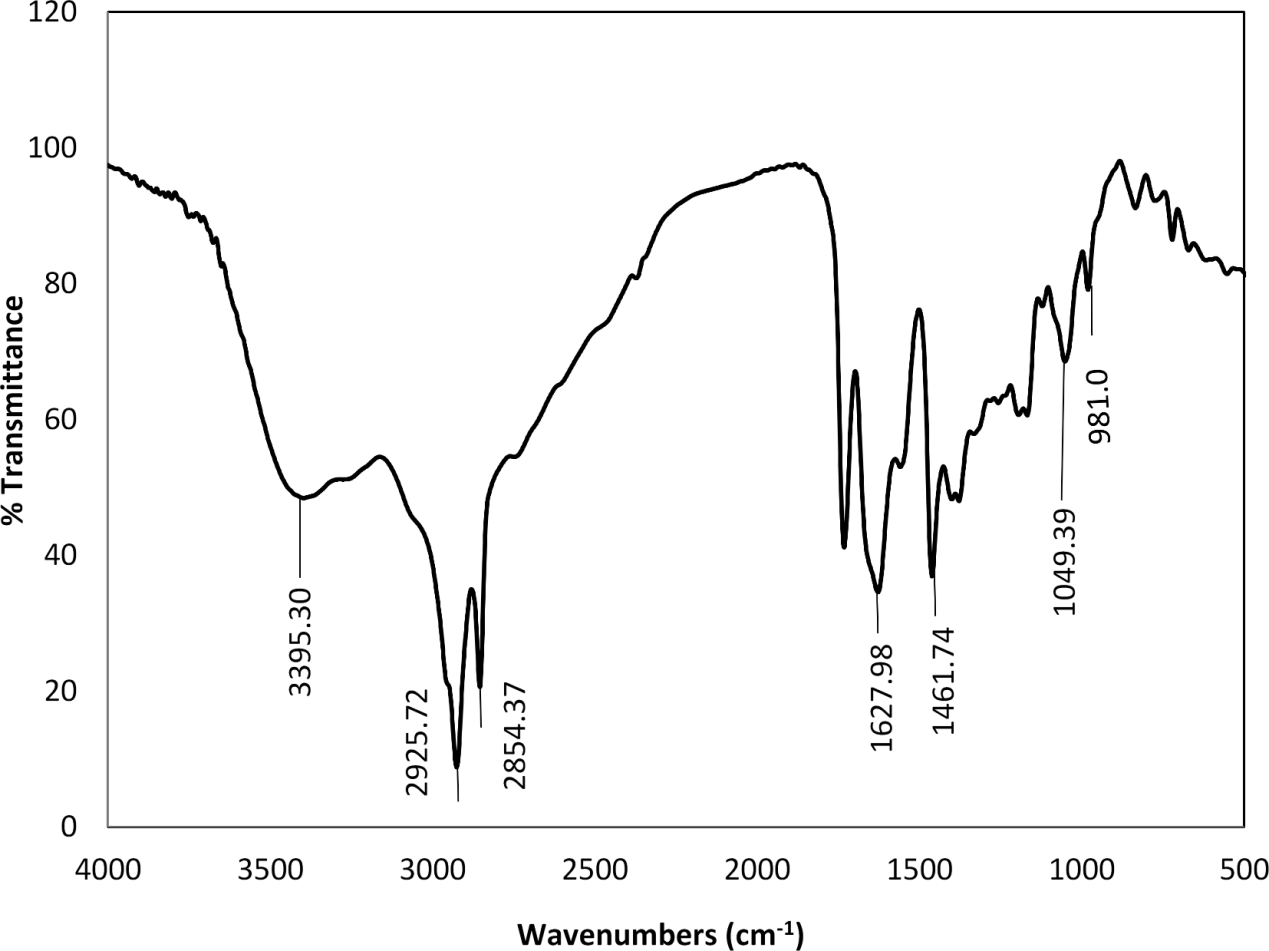
FTIR-spectrum of the prepared AAIL [G0.5 C12][Pro].

The FTIR spectrum of AAIL [G0.5 C12][Pro] exhibited a broad band at 3398.30 cm⁻^1^, corresponding to N–H stretching vibrations. Peaks at 2925.72 cm⁻^1^ and 2854.37 cm⁻^1^ were attributed to the asymmetric and symmetric stretching vibrations of CH₃ groups, respectively. The C=O stretching of the proline anion appeared at 1627.98 cm⁻^1^. A band at 1461.74 cm⁻^1^ was due to C-H bending vibrations of methylene groups (CH₂), while the peak at 1400.49 cm⁻^1^ corresponded to C-H bending vibrations of methyl groups (CH₃). The band at 1049.39 cm⁻^1^ indicated C-O stretching vibrations, indicating the presence of an ester group, and the peak at 981.07 cm⁻^1^ was associated with the C-N cation. These results confirm the successful synthesis of AAIL [G0.5 C12][Pro], as shown in Fig. 2.

### ^1^H-NMR spectra

**Fig. 3:**
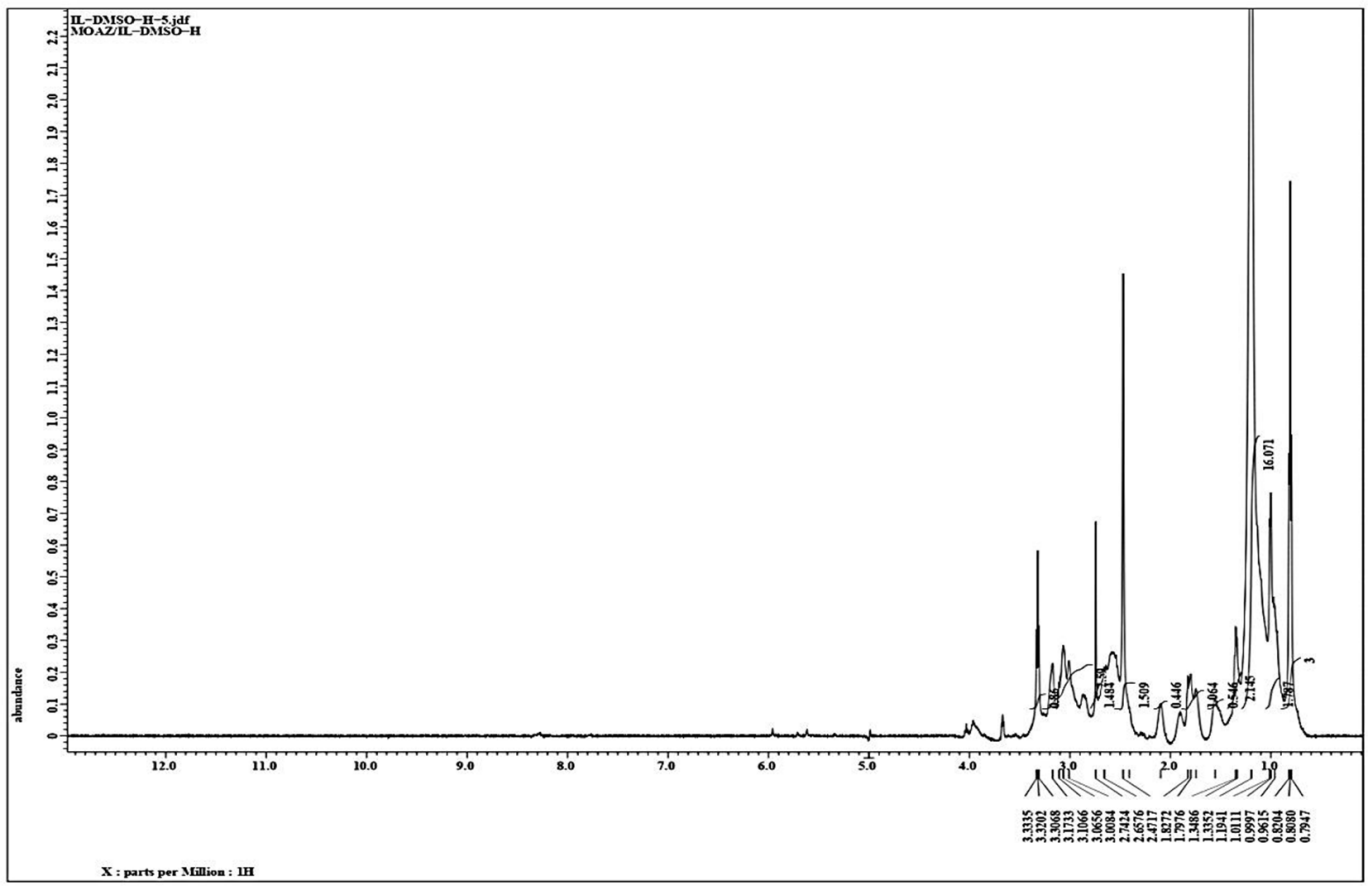
^1^H-NMR spectra of the prepared AAIL [G0.5 C12][Pro].

^1^H-NMR spectra (300 MHz, DMSO) were obtained, revealing signals at δ = 0.81 ppm for the [(CH_2_)n-CH_3_] group, δ = 1.23 ppm for additional [(CH_2_)n-CH_3_] groups, δ = 3.12 ppm for the [CH2-CH(CH_3_)-CO] group, δ = 3.21 ppm for the [CH_2_^-^N^+^] group, δ = 3.30 ppm for the [cyclic CH_2_-N +] group, δ = 3.32 ppm for the [COOCH_3_] group, and δ = 3.06 ppm for the [COO^_^] proline anion group of the amino acid. These findings align with the anticipated molecular structure as illustrated in Fig. 3. These NMR shifts corroborate the successful integration of the proline-based structure into AAIL [G0.5 C12][Pro], validating its molecular integrity as expected.

### Surface-active characteristics of the synthesized AAIL [G0.5 C12][Pro]

**Fig. 4:**
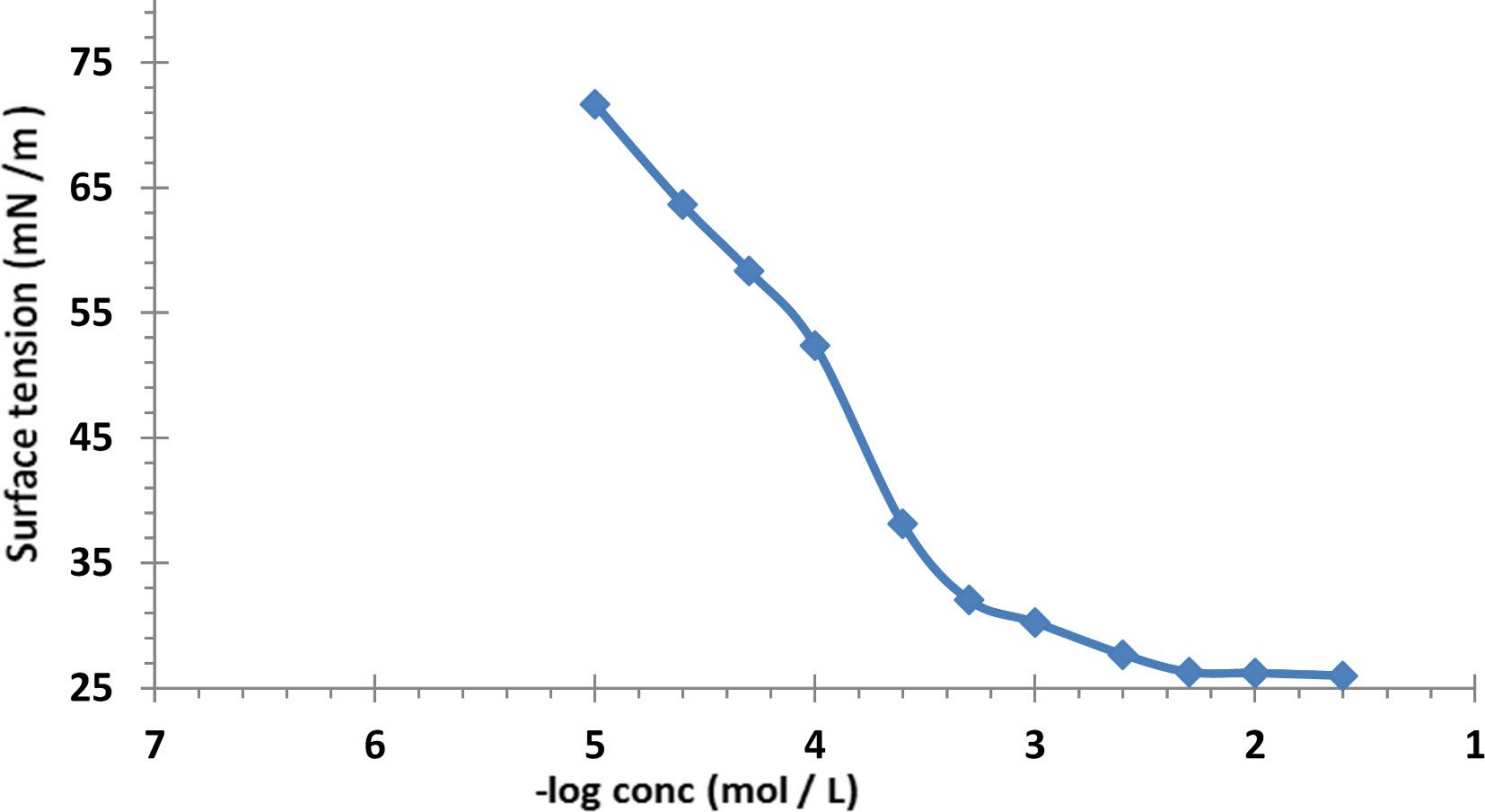
Variation of the surface tension with log concentrations for synthesized AAIL [G0.5 C12][Pro] at 25 °C.

Surface Performance Parameters of AAILs in EOR The surface-active characteristics of the synthesized AAIL [G0.5 C12][Pro] compound have been measured using traditional techniques in an aqueous medium at a temperature of 25 °C. Critical micelle concentration (cmc) and surface tension at critical micelle concentration (γcmc) were key parameters. The values of cmc and γcmc were determined using the plot’s breakpoint (Fig. 4). The surface tension (γ) decreased with increasing concentration of the produced compound, showing the adsorption of the AAIL molecule at the water–air interface. Additional surface performance parameters such as effectiveness at critical micelle concentration (Πcmc), maximum surface excess (Γmax), and minimum surface area (Amin) were also evaluated. These parameters are crucial in the context of EOR as they influence the reduction of interfacial tension between the oil and water phases, facilitating enhanced oil recovery. Table 1 summarizes the surface performance parameters of AAIL [G0.5 C12][Pro] and compares them with other conventional ILs used in EOR applications. This comparison showed that the synthesized AAIL [G0.5 C12][Pro] has *cmc* value lower than (G_0.5_ C_12_), which indicates that the studied AAIL [G0.5 C12][Pro] has an excellent ability to form micelles, due to their higher adsorption efficiency at the air–water interface and as a result higher water surface tension decrease. These results agreed with several studies suggesting that ionic liquids aggregate into smaller micelles, compared to their ammonium counterparts, with lower aggregation numbers^55^. The surface tension values at the respective cmcs (*γ*_cmc_) Table 1 showed that AAIL [G0.5C12][Pro] possesses lower surface tension at critical micelle concentration (*γ*_cmc_) than G0.5 C12.

#### Chemical phase stability tests

**Fig. 5:**
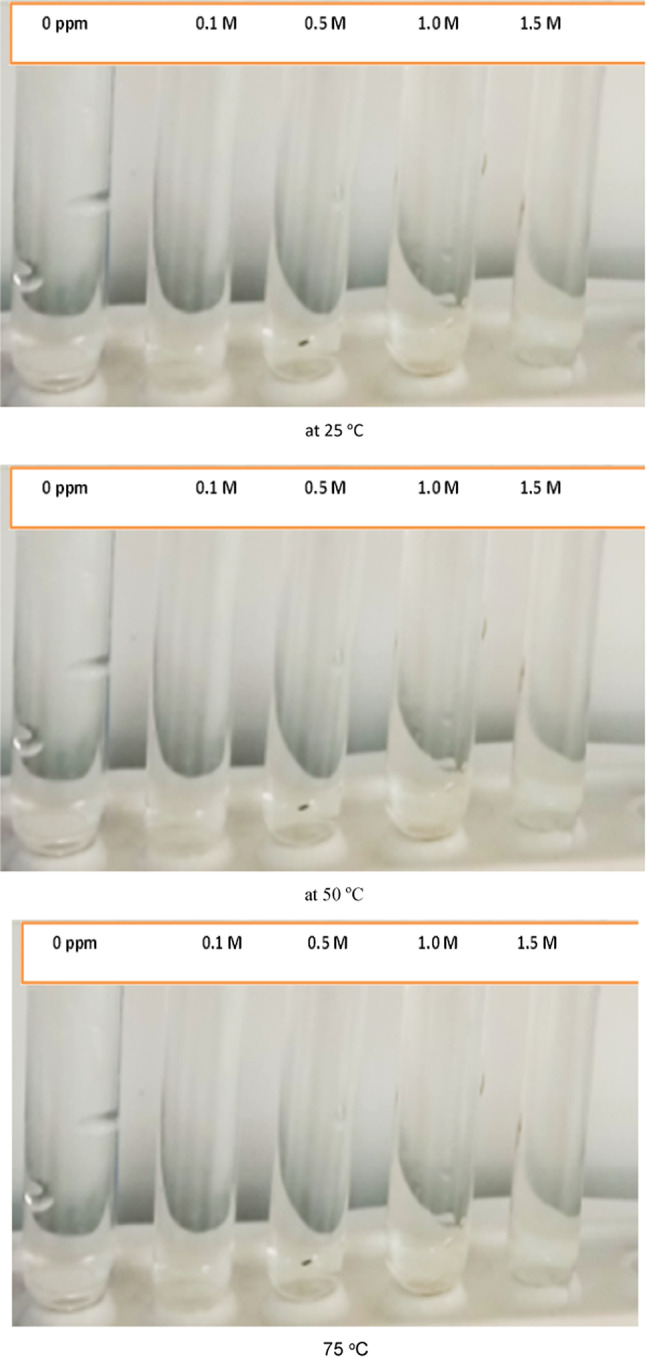
Stability of AAIL [G0.5 C12][Pro] in different salinity.

The ionic liquid [G0.5 C12][Pro] was tested for its stability in sodium chloride (NaCl) solutions of varying concentrations (0.1 M, 0.5 M, 1.0 M). The samples were observed under different temperatures (25 °C, 50 °C, 75 °C). As shown in Fig. 5, the ionic liquid remained transparent and stable in all NaCl solutions, exhibiting no visible signs of precipitation or aggregation. This indicates excellent stability of the ionic liquid in the presence of the salt solutions.

#### IFT between AAIL [G0.5 C12][Pro] and crude oil

**Fig. 6:**
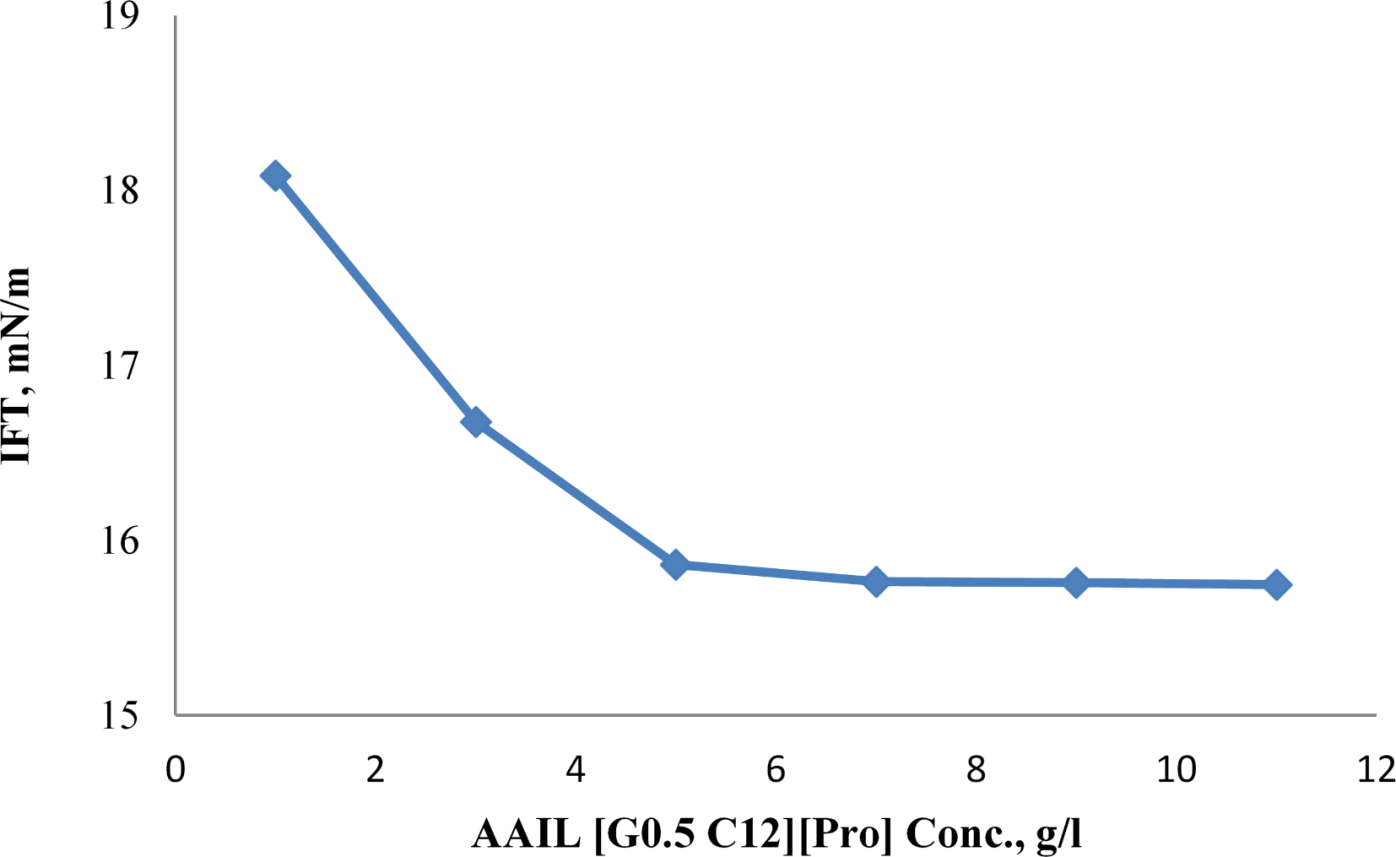
Interfacial tensions (IFTs) between AAIL [G0.5 C12][Pro] concentrations and crude oil.

The relationship between the concentrations of AAIL [G0.5 C12][Pro] and the IFT between AAIL [G0.5 C12][Pro] and crude oil is obscured in (Fig. 6). As illustrated in the figure, the IFT between AAIL [G0.5 C12][Pro] and crude oil decreases with an increase in AAIL [G0.5 C12][Pro] concentration. This means that AAIL [G0.5 C12][Pro] effectively reduces the IFT between oil and water. The figure also shows that the IFT reaches a minimum value (15.76 mN/m) at a minimum ionic liquid concentration (MIC) of about 7 g/L AAIL [G0.5 C12][Pro].

### Effect of cation salinity on interfacial tension

**Fig. 7:**
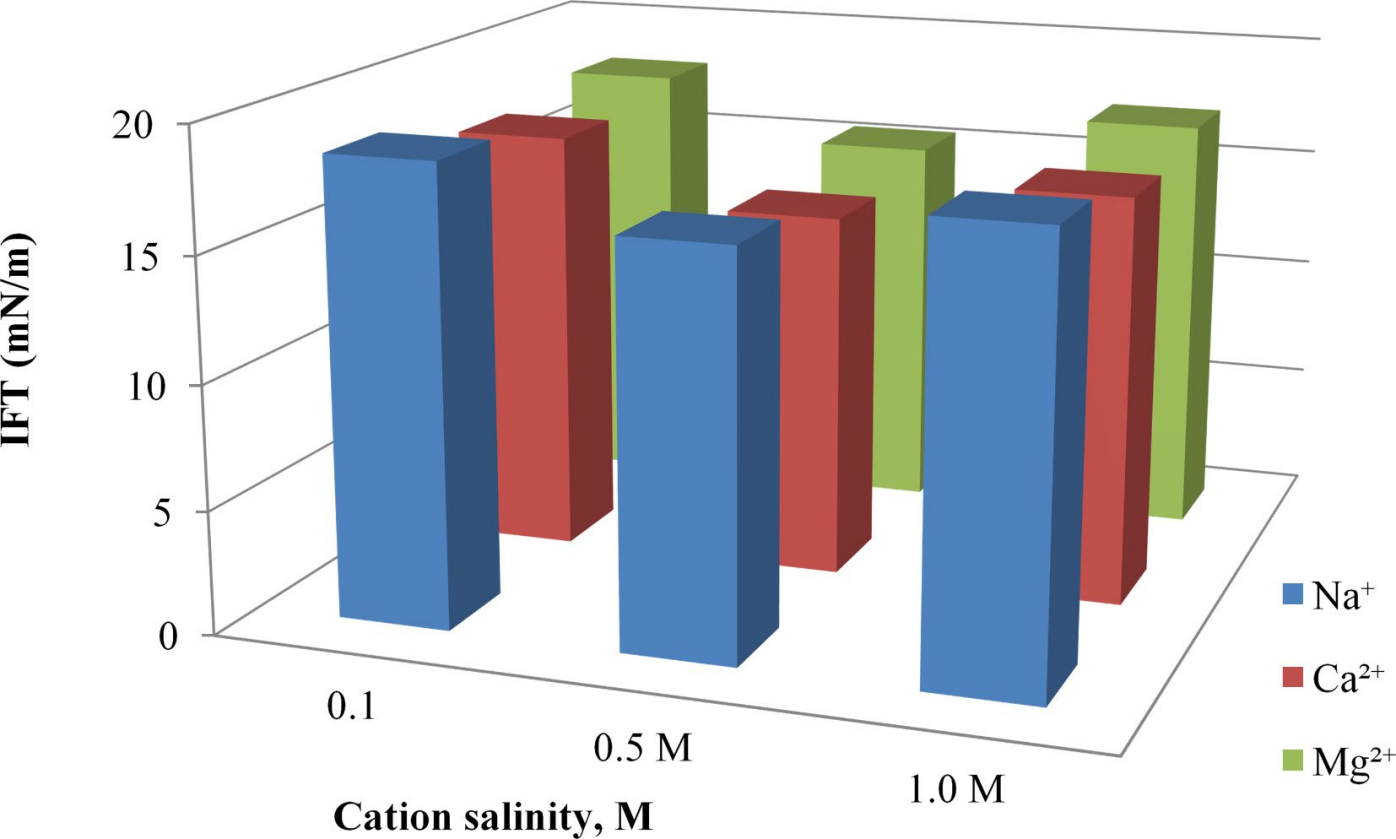
Effect of cation salinity on interfacial tension with [G0.5 C12][Pro].

Figure 7 illustrates the effect of cation salinity on the interfacial tension (IFT) of [G0.5 C12][Pro]. The results showed that the IFT of [G0.5 C12][Pro] generally decreased with increasing cation salinity for all three cations (Na^+^, Ca^2+^, and Mg^2+^). However, the specific effect of each cation on the IFT varied. At a given salinity, Ca^2+^ had the most significant impact on reducing IFT, followed by Mg^2+^ and Na+. This indicated that the interaction between the ionic liquid and the cations was influenced by the cation’s charge density and size. The stability of AAIL [G0.5 C12][Pro] in high-salinity environments (up to 1.0 M NaCl) is a critical advantage over conventional surfactants. As shown in Fig. 7, the ionic liquid maintains its interfacial activity and structural integrity across salinity levels, demonstrating its suitability for EOR applications in challenging reservoirs.

### Rheological characterizations

Results of the rheological experiment indicate a concentration-dependent behavior for viscosity, with increasing concentrations of AAIL [G0.5 C12][Pro] generally leading to higher viscosities. The relationship between concentration and viscosity should be further explored and quantified to understand the system’s behavior better. Additionally, the reproducibility of these data should be addressed to ensure the reliability of the results.

**Fig. 8:**
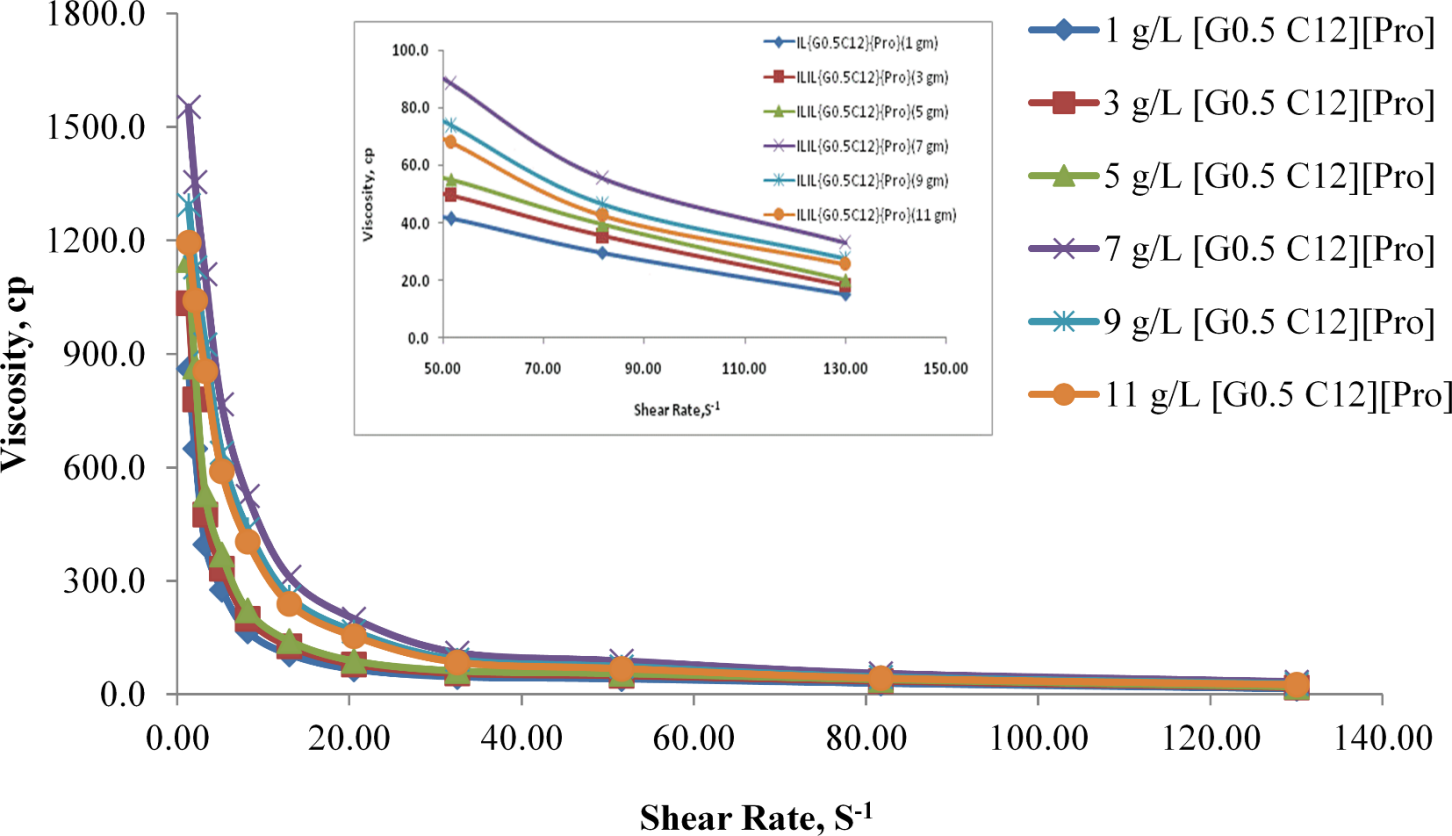
Flow curve of shear rate versus viscosity at different concentrations of synthesized AAIL [G0.5 C12][Pro].

Figure 8. provided a detailed rheological measurement analysis of the flow curve for shear rate versus viscosity and shear rate versus shear stress at different concentrations at (1, 3, 5, 7, 9, and 11 g) of synthesized AAIL [G0.5 C12][Pro]. The inverse relationship between shear rate and viscosity is typical for substances that exhibit this behavior. The increase in viscosity with higher concentrations of AAIL [G0.5 C12][Pro] aligns with expectations, as more molecules in the solution result in stronger intermolecular interactions, leading to increased resistance to flow. At lower concentrations, the intermolecular interactions between AAIL [G0.5 C12][Pro] molecules might be weaker, leading to a different shear-thinning behavior compared to higher concentrations. Specific properties of AAIL [G0.5 C12][Pro], like its charge distribution or conformation, might be more pronounced at lower concentrations, leading to distinct shear-thinning behavior. Consequently, the concentration of 7 gm was considered an optimum concentration.

**Fig. 9:**
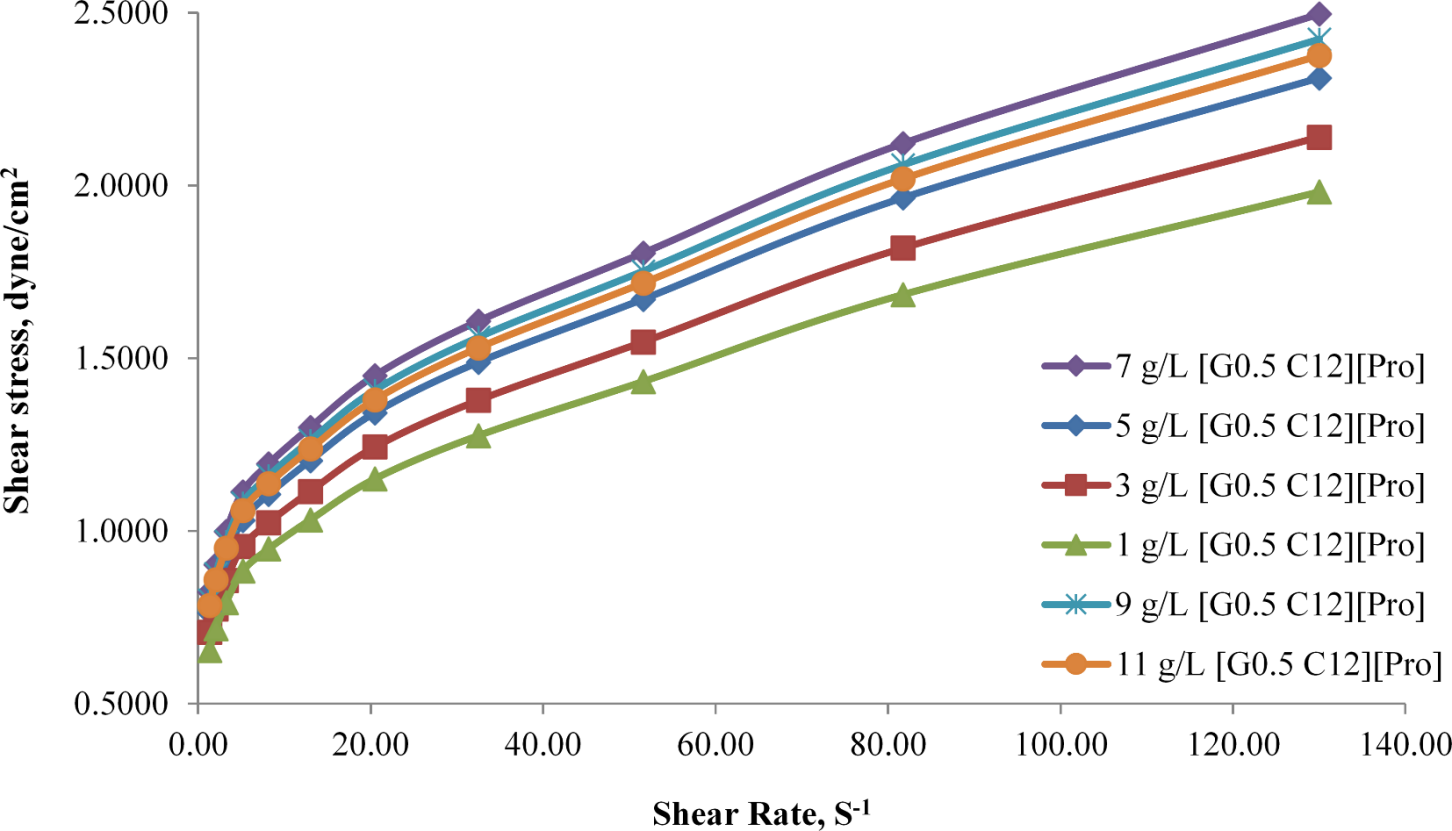
Flow curve of shear rate versus shear stress curve at different concentrations of synthesized AAIL [G0.5 C12][Pro].

Figure 9. describes the flow curve for shear rate versus shear stress for different concentrations of synthesized AAIL [G0.5 C12][Pro] (from 1 g/L to 11 g/L). This type of rheological data is commonly used to characterize the flow behavior of fluids, particularly in the context of complex fluids like ionic liquids. The general trend observed is that as the shear rate increases, shear stress also increases. This behavior indicates a non-Newtonian fluid, and the shear stress increases with shear rate, suggesting that the fluid becomes more viscous at higher shear rates. The curves demonstrate that as the AAIL [G0.5 C12][Pro] concentration increases, the shear stress also increases. This implies that the viscosity of the fluid is directly influenced by the concentration of the AAIL [G0.5 C12][Pro]. Higher concentrations result in higher shear stress, indicating a more viscous fluid. The specific example "IL [G0.5 C12][Pro] (7 g)" suggests that the curve represents the AAIL [G0.5 C12][Pro] solution with a concentration of 7 g. This curve has the highest shear stress among the ones presented.

### Effect of temperature on synthesized AAIL[G0.5C12][Pro] viscosity

**Fig. 10:**
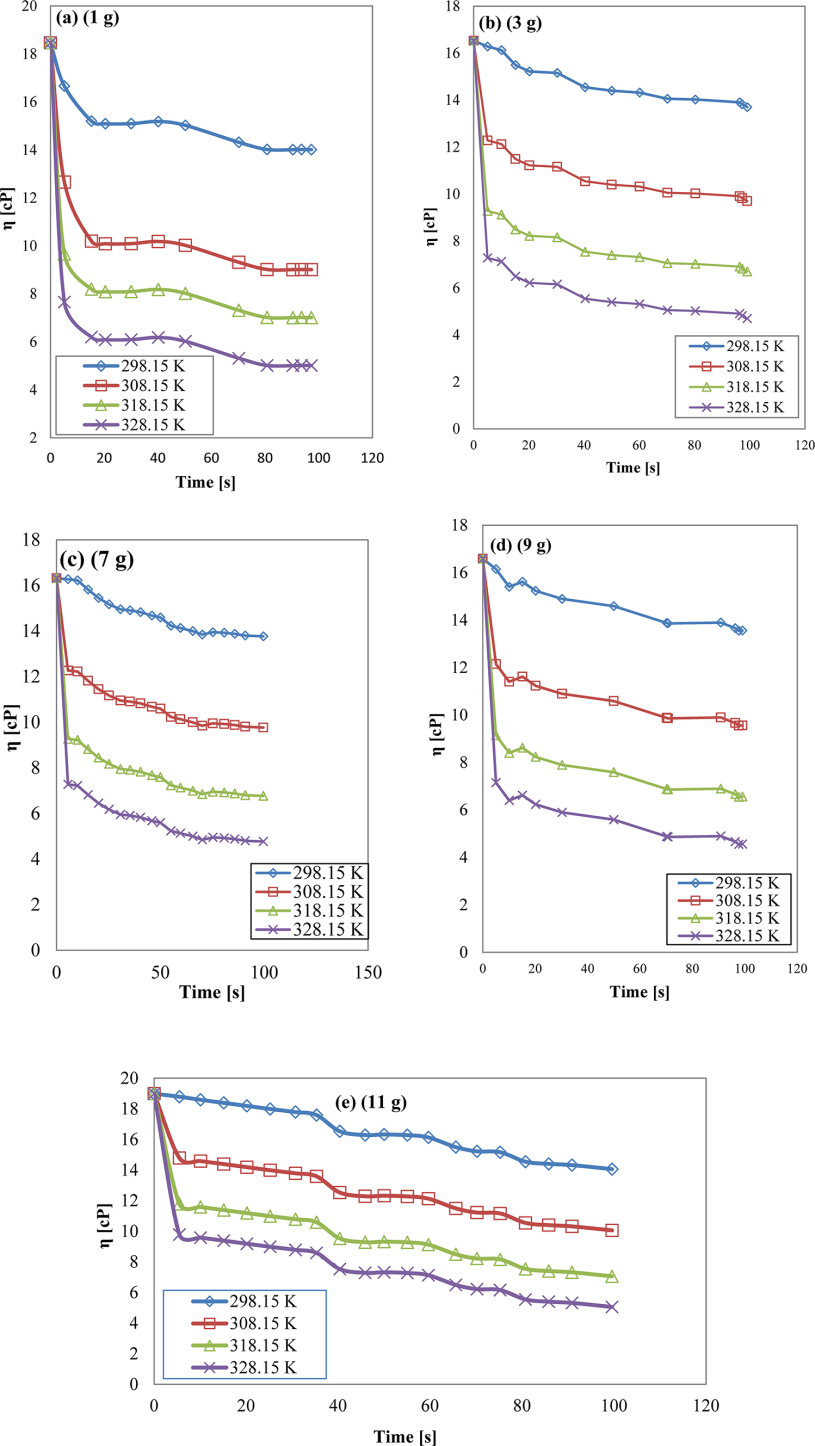
Viscosity virous time at differents temperatures for synthesized AAIL [G0.5 C12][Pro] (**a**) (1 g), (**b**) (3 g), (**c**) (7 g), (**d**) (9 g) and (**e**) (11 g).

Figure 10a–e illustrates the temperature-dependent viscosity of the synthesized AAIL [G0.5 C12][Pro] at various concentrations (1, 3, 5, 7, 9, and 11 g). The observed trend in all concentrations shows a decrease in viscosity as the temperature increases. As the temperature increases, the ionic liquid chains gain more energy, overcoming intermolecular forces that hinder their movement. This results in a lower viscosity, indicating that the liquid becomes more fluid and flows more easily. The systematic exploration of these trends across different concentrations provides valuable insights into the temperature-dependent behavior of the synthesized AAIL^56^.

### Wettability Alteration through Amino Acid Ionic Liquids

**Fig. 11:**
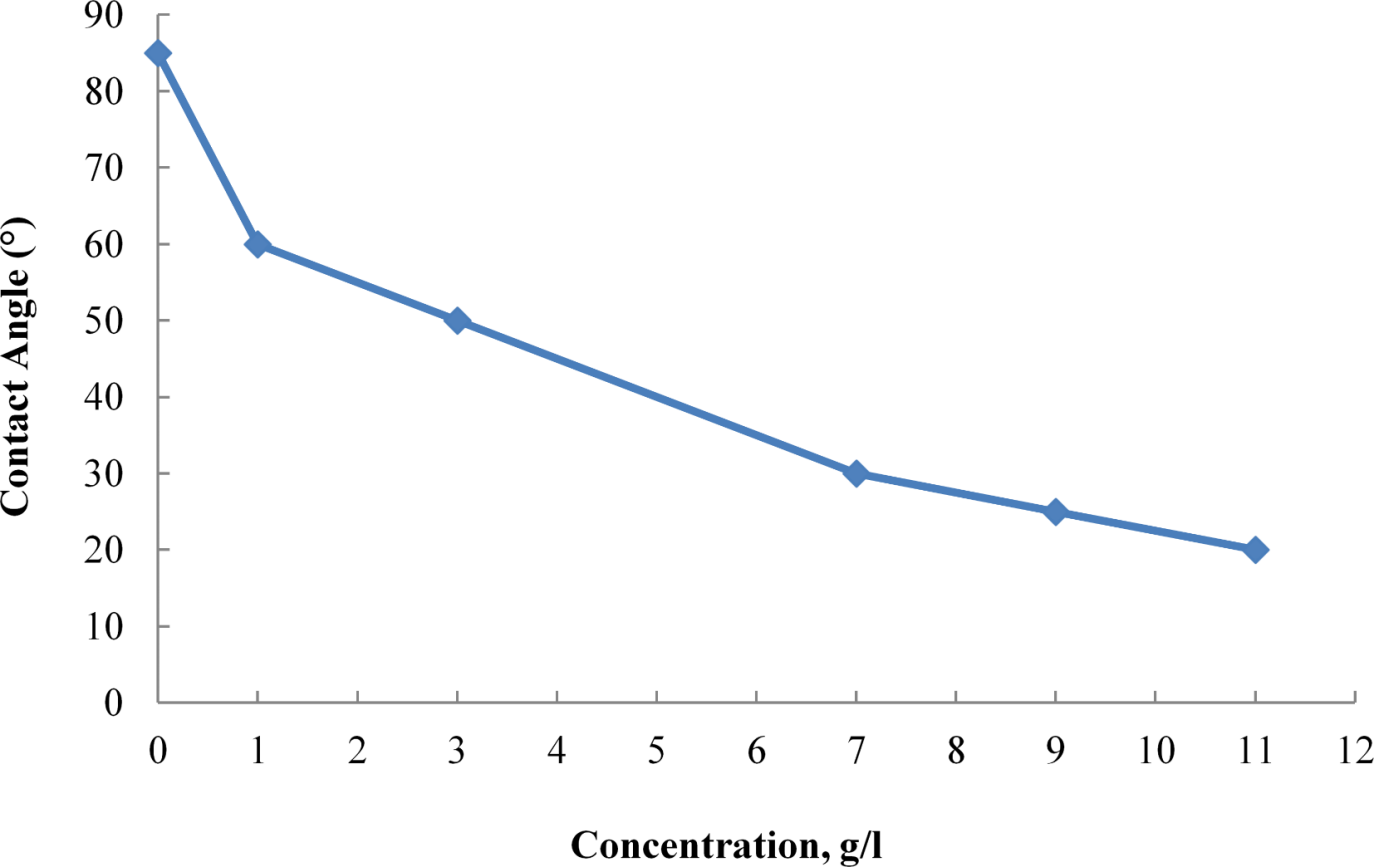
Effect of [G0.5 C12][Pro] Concentration on Contact Angle of Reservoir Rock.

Figure 11 illustrates the relationship between the concentration of the amino acid ionic liquid [G0.5 C12][Pro] and the measured contact angle on reservoir rock surfaces. As the concentration of [G0.5 C12][Pro] increases, there is a marked reduction in the contact angle, indicating a transition towards water-wet conditions.

At 7 g/L, the contact angle decreases to 30°, reflecting a significant alteration in wettability that enhances oil mobilization. The data demonstrates that the optimal concentration for achieving effective wettability modification lies around this level, which facilitates improved interaction between the ionic liquid and the rock surface. This shift in wettability is crucial, as it directly correlates with the ionic liquid’s ability to improve oil recovery rates.

The continued decrease in contact angle at higher concentrations, such as 9 g/L and 11 g/L, further reinforces the effectiveness of [G0.5 C12][Pro] as a wettability modifier. However, it is important to note that while increasing concentration enhances the wettability change, practical applications must consider the optimal concentration that maximizes oil recovery without introducing excessive costs or environmental concerns. Thus, the results from Fig. 9 underscore the potential of [G0.5 C12][Pro] as a viable agent for enhancing oil recovery through effective wettability alteration.

The moderate reduction in IFT achieved by AAIL [G0.5 C12][Pro] (15.57 mN/m) is complemented by its strong wettability alteration capabilities. Contact angle measurements show a reduction from 105° to 30°, indicating a significant shift from oil-wet to water-wet conditions. This combined effect of IFT reduction and wettability alteration contributes to enhanced oil mobilization, effectively addressing capillary pressure limitations.

### Adsorption of AAIL [G0.5 C12][Pro] on the solid phase (rock surface)

The adsorption behavior of AAIL [G0.5 C12][Pro] on the rock surface plays a pivotal role in altering wettability and enhancing oil recovery efficiency. As highlighted by the contact angle measurements in this study, the presence of AAIL [G0.5 C12][Pro] leads to a marked reduction in contact angle, shifting the surface from oil-wet to water-wet conditions. This wettability alteration is critical for improving oil displacement and recovery. At a concentration of 7 g/L, the contact angle was reduced to 30°, confirming the effective adsorption of AAIL [G0.5 C12][Pro] onto the rock surface. Thermodynamic analysis further supports this observation, revealing a highly favorable ΔGads value of -22.3 kJ/mol (Table 1). This negative value indicates spontaneous and strong adsorption, which reduces oil adhesion on rock surfaces and facilitates oil mobilization. These results underscore the capability of AAIL [G0.5 C12][Pro] to modify rock-fluid interactions and enhance oil displacement effectively.

### Advantages and disadvantages of [G0.5 C12][Pro]

The amino acid ionic liquid [G0.5 C12][Pro] presents several advantages that make it a promising candidate for enhanced oil recovery (EOR). Its environmentally friendly nature, derived from naturally occurring amino acids, positions it as a sustainable alternative to conventional surfactants, potentially reducing the environmental impact associated with oil recovery processes. Additionally, this ionic liquid has demonstrated significant potential in enhancing oil recovery through effective wettability modification and substantial reduction of interfacial tension, which leads to improved oil mobilization in reservoir rocks. The tunable properties of [G0.5 C12][Pro], which can be adjusted by varying its concentration, allow for optimization based on specific reservoir conditions, enhancing its performance in diverse environments. Furthermore, preliminary results indicate that [G0.5 C12][Pro] maintains stability under elevated temperatures and salinities, making it suitable for application in challenging reservoir conditions.

However, there are notable disadvantages associated with the use of [G0.5 C12][Pro]. The synthesis of this amino acid ionic liquid has primarily been conducted at a laboratory scale, raising concerns about the scalability of the production process for industrial applications, which may present economic and logistical challenges. Limited data on the long-term stability of [G0.5 C12][Pro] in the presence of crude oil and varying reservoir conditions necessitate further research to assess its efficacy over time. Additionally, while laboratory experiments show promising results, comprehensive field testing is required to confirm its practicality in real-world applications. Lastly, the potential interactions of [G0.5 C12][Pro] with other additives commonly used in EOR processes remain to be explored, highlighting the need for additional investigation to optimize formulations and maximize recovery outcomes.

### Flooding test

The AAIL [G0.5C12][Pro] solution could enhance the oil’s displacement efficiency, ultimately chemically enhanced oil recovery. It improves the displacement efficiency of the oil by reducing the interfacial tension between the oil and water and altering the reservoir wettability^2^.

**Fig. 12:**
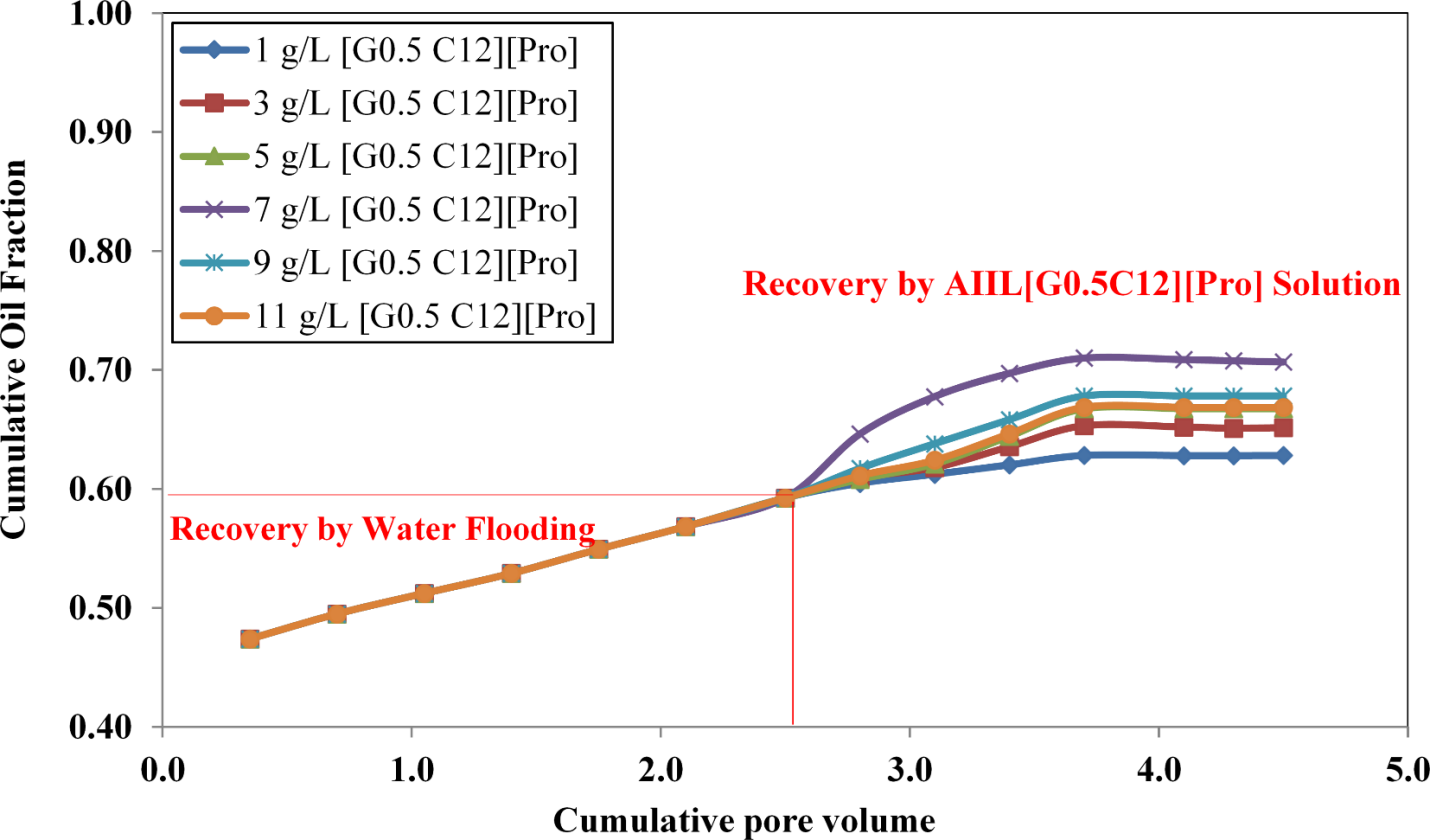
Oil recovery by water and synthesized AAIL [G0.5 C12][Pro] solution.

The flooding test was performed under the reservoir conditions up to five times the pore volume (5 PV) through different stages using a ¼ five-spot sand pack model, see Fig. 12. All the core flooding displacements were performed on the 2D-Sand pack model (1/4 Five-Spot); this model has 2,700 cc bulk volume (_*Vb*_) and 607.5 cc pore volume (*Pv*)*,* which gives a porosity of 22.5%^57^. These displacement tests were performed at a reservoir pressure of 1,500 psi and temperature of 167 °F. The reservoir oil has the following characteristics in Table 2.

**Table 2:** The reservoir oil characteristics.

Oil gravity	34 ^o^API
Oil viscosity	1.726 cP
GOR	23.11 SCF/STB
Bubble point (BP)	95 psi

The displacements run to condition the sand pack model before conducting the flooding displacement; the initial oil volume (*V*_*oi*_) is about 472 cc, which is equivalent to an initial oil saturation (*S*_*oi*_) of 77.8%, while the initial water saturation (*S*_*wi*_) at 22.2% and the residual oil saturation (*S*_*or*_) at 31.7%^58^. The results of these flooding tests are shown in Fig. 12. as the following:

1. Water flooding achieves oil recovery of around 60% at 2.5 PV.
2. Then IL [G0.5 C12][Pro] solution flooding with different concentrations (from 1 to 11) started at 2.5 PV till it reached 4.7 PV, which achieves oil recovery up to 71%.

The AAIL[G0.5 C12][Pro] solution enhances the oil recovery by giving an incremental recovery of 11% (60%—71%) compared to water flooding.

**Fig. 13:**
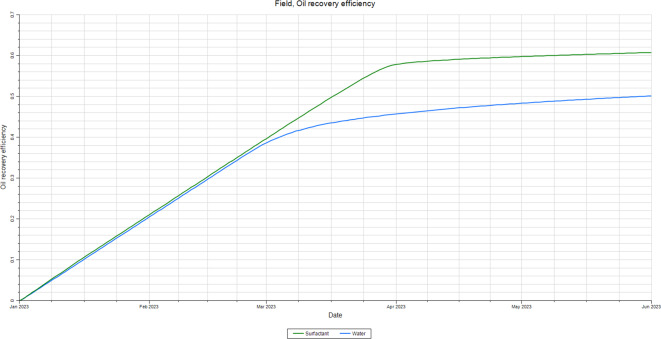
Oil Recovery for water flooding and synthesized AAIL [G0.5 C12][Pro] flooding.

They are using reservoir simulation to check the results of the flooding test performed in the lab^59^. Petrel and Eclipse are the most used simulation software in the oil industry worldwide, and they are used to build the reservoir model and run the flooding test simulations. The static model was constructed in Peterel, and Eclipse was used to run the simulation calculations^60^. The simulation results are shown in Fig. 13. The oil recovery from the water flooding is about 50%, while the oil recovery from the ionic liquid flooding is about 61%. It’s about 11% incremental gained for the ionic liquid case more than the water flooding case^61^.

**Fig. 14:**
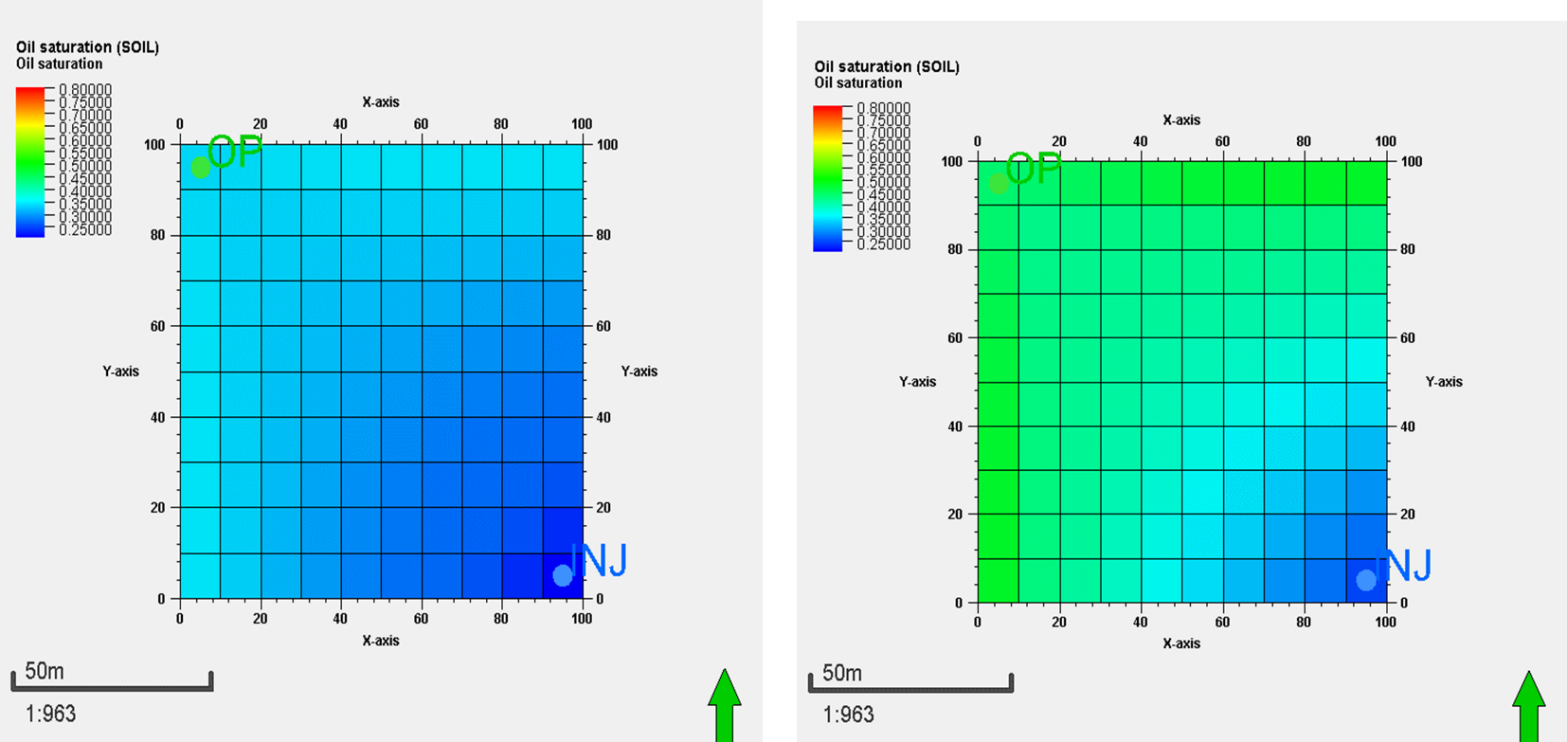
Left is the oil saturation at the end of the synthesized AAIL [G0.5 C12][Pro] flooding; Right is the oil saturation at the end of the water flooding.

The ionic liquid simulation confirms better displacement efficiency compared to water flooding. Figure 14. shows both cases’ oil saturation at the end of the flooding. The oil saturation at the end of the simulation time of the water flooding on the right, while the oil saturation of the ionic liquid on the left. It’s clear the displacement efficiency of the ionic liquid is much better than the water flooding as the oil saturation at the end of the simulation time of the ionic liquid case is much lower than the water flooding case^62^. It’s clear that the ionic liquid has less remaining oil saturation compared to the water flooding due to better displacement and sweep efficiency^63^.

### Comparative EOR performance of AAIL [G0.5 C12][Pro] with various ionic liquids

The comparison Table 3 highlights the performance of AAIL [G0.5 C12][Pro] in Enhanced Oil Recovery (EOR) applications, contrasting it with various ionic liquids documented in the literature. This study achieved a remarkable (11% IOOP) increase in oil recovery under laboratory conditions and in reservoir simulations. In comparison, Bin Dahbag et al. (2015) reported a 5% increase in oil recovery using Ammoeng 102 IL^64^, while Alarbah et al. (2017) demonstrated an 8.77% increase using 1-Ethyl-3-Methyl-Imidazolium Acetate ([EMIM][Ac])^65^. Rodríguez-Palmeiro et al. (2017) recorded an 8.10% increase in Tributyl (tetradecyl) phosphonium chloride ionic liquid^66^, and Liu et al. (2018) achieved enhancements of 55–60% using phosphonium-based ionic liquids^67^. Despite the promising results of these previous studies, the performance of AAIL [G0.5 C12][Pro] surpasses them, underscoring its superior efficacy in EOR processes and its potential for practical application.

**Table 3:** EOR performance comparison: AAIL [G0.5 C12][Pro] vs. other studies.

Ionic liquid type	Conditions	Aditional oil recovery (%IOOP)	Refs
Ammoeng 102	Sandstone reservoir; conventional water flooding	5%	^64^
[EMIM][Ac]	Medium oil reservoir; high salinity	8.77%	^65^
4000 ppm [P_4 4 4 14_]Cl + 5000 ppm NaOH	Carbonate reservoir; water flooding	Up to 10%	^66^
Phosphonium-based	High-salinity reservoir; HPHT conditions	5%	^67^
AAIL [G0.5 C12][Pro]	Sandstone and carbonate reservoirs 75 °C, 1500 psi	11%	This Study

Compared to other ionic liquids and surfactants used in EOR, AAIL [G0.5 C12][Pro] demonstrates superior performance under high salinity and elevated temperatures. The combination of moderate IFT reduction, strong wettability alteration, and stability under extreme conditions positions AAIL [G0.5 C12][Pro] as a promising candidate for challenging reservoirs.

### Challenges and limitations

While this study successfully synthesizes and evaluates the amino acid ionic liquid [G0.5 C12][Pro] for enhanced oil recovery (EOR), several challenges and limitations should be acknowledged.

1. The synthesis of AAIL [G0.5 C12][Pro] using quaternary ammonium salt PAMAM G0.5 C12 and proline is currently conducted at a laboratory scale. The scalability of this synthesis process for industrial applications remains to be investigated, as large-scale production may present economic and logistical challenges.
2. The experiments were conducted under controlled laboratory conditions, which may not fully replicate the complex environments found in actual oil reservoirs. Factors such as varying temperature, pressure, and salinity may influence the performance of AAIL [G0.5 C12][Pro], and further studies are needed to evaluate its efficacy under diverse reservoir conditions.
3. The long-term stability of the amino acid ionic liquid in the presence of crude oil and reservoir conditions has yet to be extensively studied. Future research should address how prolonged exposure to harsh conditions affects the performance and stability of [G0.5 C12][Pro].
4. The interaction of [G0.5 C12][Pro] with other additives and chemicals commonly used in EOR processes has yet to be assessed. Understanding these interactions is crucial for optimizing formulations and achieving the best recovery outcomes.
5. Although the operational costs of ionic liquids like [G0.5 C12][Pro] are higher than conventional surfactants, the incremental oil recovery (11%) observed in this study justifies their application in reservoirs where traditional methods are ineffective. Furthermore, the environmentally friendly nature of [G0.5 C12][Pro] offers an added advantage, aligning with industry goals to reduce the environmental impact of EOR processes. A cost–benefit analysis under field-scale conditions is recommended for future studies to further evaluate economic viability."

By recognizing these challenges and limitations, this study aims to provide a balanced view of the potential applications of AAIL [G0.5 C12][Pro] while highlighting the need for further research to realize its benefits in enhanced oil recovery fully.

## Conclusion

This study focuses on the synthesis and evaluation of the amino acid ionic liquid AAIL [G0.5 C12][Pro] as an effective agent for enhanced oil recovery (EOR). This research provides valuable insights and supports the practical application of AAIL [G0.5 C12][Pro] in EOR processes, demonstrating its effectiveness in improving oil recovery rates through several vital contributions:

1. The innovative synthesis of AAIL [G0.5 C12][Pro] using quaternary ammonium salt PAMAM G0.5 C12 and proline, confirmed by FTIR and 1H-NMR analyses.
2. Key findings include spontaneous adsorption of AAIL molecules at the air–liquid interface, as indicated by negative ΔGads values, and concentration-dependent, non-Newtonian rheological behavior. Viscosity decreases with increasing temperature, enhancing flow properties.
3. These results align with previous research on the temperature-dependent viscosity of ionic liquids, confirming that increased temperature reduces intermolecular forces and facilitates movement.
4. The experimental conditions were optimized by varying the concentration of AAIL [G0.5 C12][Pro] and evaluating its performance at different temperatures. The maximum oil recovery was achieved at a concentration of 7 g and optimal temperature conditions identified through systematic variation and performance assessment.
5. Laboratory sand-pack experiments showed a 65% increase in oil recovery, and reservoir model simulations demonstrated a 61% increase, highlighting the practical EOR potential of AAIL [G0.5 C12][Pro].

The results from this study highlight the dual mechanisms of action—moderate IFT reduction and strong wettability alteration—by which AAIL [G0.5 C12][Pro] enhances oil recovery. Its stability in high salinity and high-temperature environments further underscores its potential for application in challenging reservoirs. Future work will focus on scaling up the synthesis process, conducting field trials, and exploring the economic feasibility of this ionic liquid for large-scale EOR applications.

## Electronic supplementary material

Below is the link to the electronic supplementary material.

Supplementary Material 1

## Data Availability

The raw data used and analyzed during the current study is available from the corresponding author upon reasonable request.

## Abbreviations

EOR: Enhanced oil recovery
Cmc: Critical micelle concentration
Γcmc: Surface tension at critical micelle concentration
Πcmc: Effectiveness at critical micelle concentration
Γmax: Maximum surface excess
Amin: Minimum area
ΔG_mic_: Standard free energies of micellization
ΔG_ads_: Standard free energies of adsorption
AAIL: Amino acid ionic liquid
S_wi_: Initial water saturation
S_oi_: Initial oil saturation
S_or_: Residual oil saturation
V_oi_: Initial oil volume
PV: Pore volume
Vb: Bulk volume
BP: Bubble point
IOOP: Incremental Oil Over the Original Oil in Place
GOR: Gas oil ratio
